# Dietary *Bacillus subtilis* benefits meat quality by regulating the muscle fiber type and antioxidant capacity of broilers

**DOI:** 10.1016/j.psj.2024.104267

**Published:** 2024-08-25

**Authors:** Hairong Wang, Chuanpi Xiao, Jiqiang Li, Rongrong Liang, Yunge Liu, Zhigang Song, Johan Buyse, Lixian Zhu

**Affiliations:** ⁎ College of Food Science and Engineering, Shandong Agricultural University, Tai'an, Shandong, 271018, China; † College of Animal Science and Technology, Shandong Agricultural University, Tai'an, Shandong, 271018, China; ‡ Division Laboratory of Livestock Physiology, Department of Biosystems, Leuven 3001, Belgium

**Keywords:** probiotics, fiber transformation, AMPK, Nrf2

## Abstract

The effects of dietary *Bacillus subtilis* (**BS**) on the meat quality of broilers were evaluated, with an emphasis on the regulation of muscle fiber types and antioxidant capabilities. One hundred and forty-four Arbor Acres male broilers were divided into 3 treatment groups (0, 300 mg/kg and 500 mg/kg dietary BS) and raised for 35 d. The results suggested that BS improved meat quality by improving the muscular pH, meat color, water holding capacity and shear force. Immunofluorescence staining revealed a positive impact of BS on the muscle fiber transformation in thigh muscles, and the gene/protein expression data from specific muscle fiber types confirmed this finding. BS activated AMP-activated protein kinase (**AMPK**), silent information regulator 1 and peroxisome proliferator-activated receptor gamma coactivator 1alpha. The postmortem analysis revealed that BS increased the activity of glutathione peroxidase and total antioxidant capacity while decreasing the malondialdehyde content. Additionally, BS increased the gene and protein expression of nuclear factor-like 2 (**Nrf2**) and activated the Nrf2 signaling pathway, including its downstream factors, such as heme oxygenase-1, catalase, superoxide dismutase and glutathione peroxidase. In conclusion, dietary BS improved meat quality by modifying muscle fiber types and enhancing the antioxidant capacity in broilers.

## INTRODUCTION

Muscle fibers can be categorized into fast-twitch/slow-twitch (type II/type I) types and play important roles in determining the textural and sensory attributes of meat (Lee et al., 2018; Lorenzo et al., 2013; Matarneh et al., 2021). Type II fibers are associated with rapid and forceful contractions to produce tough meat; type I fibers are involved in sustained and low-intensity activities to generate tender and juicier meat (Maglischo, 2015). The distributions and proportions of these fiber types influence meat quality (Huo et al., 2022). Muscle fiber transformation involves the intricate interplay of various molecular pathways and regulatory proteins (Zhang et al., 2022), including AMP-activated protein kinase (**AMPK**), sirtuin 1 (**SIRT1**), and peroxisome proliferator-activated receptor gamma coactivator 1alpha (**PGC-1α**). AMPK plays crucial roles in maintaining cellular energy homeostasis by regulating metabolic pathways (Chaube and Bhat, 2016; Ke et al., 2018). AMPK can regulate PGC-1α to control slow-twitch muscle fiber formation (Xu et al., 2020) and increase the transcription of PGC-1α by increasing NAD^+^ levels and upregulating SIRT1 expression (Matarneh et al., 2021). In muscle tissue, the transition of type II fibers to type I fibers was promoted by AMPK activation, which are associated with an increased oxidative capacity and improved meat tenderness (Guo et al., 2022). The antioxidant capacity is another crucial factor affecting meat quality. Oxidative stress can lead to lipid peroxidation and compromise meat quality (Amaral et al., 2018; Xing et al., 2019). SIRT1 plays a key role in the regulation of mitochondrial function and oxidative stress responses (Xie et al., 2020). SIRT1 activation is linked to type I fiber promotion through its interaction with PGC-1α, contributing to improved meat quality (Zhang et al., 2023). Increased PGC-1α expression can be induced by AMPK and SIRT1 activation, thereby regulating skeletal muscle metabolism (Kong et al., 2022). PGC-1α works in concert with both AMPK and SIRT1 for muscle fiber transformation to regulate meat quality (Coppi et al., 2021).

*Bacillus subtilis* is widely recognized for its probiotic properties and ability to increase livestock health and performance (Ramlucken et al., 2020; Luise et al., 2022). Previous reports have highlighted its possible impact on the quality of meat by regulating fiber types and the antioxidant capacity (Bai et al., 2016; Cramer et al., 2018; Nie et al., 2022; Dong et al., 2023a). The antioxidant properties of BS play a vital role in mitigating oxidative damage, thereby preserving meat quality and extending its freshness (Cramer et al., 2018; Mohammed et al., 2024).

In broilers, the majority of studies on *B. subtilis* have focused on production, immune responses, and endocrinology parameters under standard conditions (Boroojeni et al., 2018) or stressful stimuli, such as high temperature and bacteria exposure (Wang et al., 2018; Abudabos et al., 2019). However, the exact pathway through which BS influences muscle fiber conversion as a dietary supplement was unknown. Therefore, the impacts of dietary *B. subtilis* on the meat quality, muscle fiber type transformation and antioxidant capacity in broilers were analyzed in the current experiment.

## MATERIALS AND METHODS

### Animals and Treatments

This experiment followed the ethical guidelines issued by the Animal Care Committee of Shandong Agricultural University (No. SDAUA-2022-50). One hundred and forty-four Arbor Acres broilers (male, 1-day-old, 43.1 ± 0.37 g; Dabao Company, Taian, Shandong, China) were randomly allocated to 3 treatment groups, with 6 replicates of 8 individuals. The broilers were fed a diet (Table 1) without BS (control group, **CON**), with low-dose BS (**LBS**, 300 mg/kg dietary BS), or with high-dose BS (**HBS**, 500 mg/kg dietary BS). The probiotic BS (DSM32324-32325), with a viable count of 3.2 × 10^9^ CFUs/g, was supplied by Chr. Hansen Trading Company (Beijing, PRC). The pellet form diet was formulated according to the “Compound Feed for Egg Laying Chickens and Broilers” (GB/T 5916-2020) in the People's Republic of China. The broilers were raised in a sealed room with environmental control equipment and ad libitum access to water and feed. The light period consisted of 23 h of light and 1 h of darkness.

**Table 1:** The ingredients of the basal diet and nutritional levels used in the experiment.

Items	1-21 d	22-35 d
Ingredient (%)		
Corn	60.00	65.33
Soybean meal (43%)	28.80	27.81
Corn protein flour (60%)	5.30	2.70
Salt	0.16	0.18
Baking soda	0.20	0.20
Limestone	1.30	1.1
Dicalcium phosphate	0.75	1.2
Soybean oil	2.20	4.10
Vitamin premix^1^	0.03	0.03
Mineral premix^2^	0.20	0.2
Choline chloride (50%)	0.1	0.1
Methionine	0.23	0.15
Lysine (70%)	0.58	0.70
Threonine (98.5%)	0.134	0.20
Phytase (20,000 U)	0.02	0.02
Total	100	100
Nutritional level		
Metabolizable energy	2850 (kcal/kg)	3050 (kcal/kg)
Crude protein	21.5	19.5
Lysine	1.26	1.19
Methionine	0.57	0.55
Calcium	0.60	0.72
Total phosphorus	0.60	0.56
Available phosphorus	0.40	0.36

1 Provided per kilogram of compound in the diet: vitamin A, 12,000 IU; vitamin D3, 5,500 IU; vitamin E, 60 mg; VK, 3.2 mg; vitamin B1, 3.2 mg; vitamin B2, 8.6 mg; nicotinic acid, 65 mg; pantothenic acid, 20 mg; vitamin B6, 4.3 mg; biotin, 0.22 mg; folic acid, 2.2 mg; and vitamin B12, 0.017 mg.

2 Provided per kilogram of compound in the diet: I, 1.25 mg; Fe, 20 mg; Mn, 120 mg; Se, 0.3 mg; and Zn, 110 mg.

### Sample Collection

At 35 d of age, 6 broilers were selected from each treatment group and slaughtered via cervical dislocation. Samples for assessments of antioxidant enzyme activity, muscle fiber composition, and molecular indices were collected from the breast and thigh muscles on the left side and stored in liquid nitrogen. The right thigh and breast muscles were removed and stored at 0 to 4°C to measure meat quality.

### Meat Quality Analysis

Five indices were determined to investigate the effects of BS on the quality of meat, including the pH value, color difference, drip/cooking loss percentage, and shear force value.

A pH meter (Seven2Go-S2, Mettler-Toledo, Switzerland) was used to measure the pH_45min_ and pH_24h_ values of the samples. Three different locations were randomly selected for the determination for each sample, and the average pH value was calculated.

Luminance (L*), yellowness (b*) and redness (a*) of the meat were determined with a colorimeter (Model SP62, Grand Rapids, MI) at 24 h *postmortem*. Three different positions on the sample were chosen at random for measurement, and the means were calculated for statistical analysis.

Drip loss was determined with a modified protocol from the literature (Jin et al., 2021a). Approximately 10 ± 0.5 g of each sample (**M1**) was used for the measurement. The samples were suspended in paper cups with a wire, incubated at 4°C for 24 h, and then, weighed a second time (**M2**) after blotting with filter paper to absorb the surface water. The ratio of drip loss was analyzed with the formula “(M1-M2)/M1 × 100”.

Cooking loss was conducted according to the protocol of Dong et al. (2023b). Briefly, the sample was weighed before cooking (W1, initial weight), packed in plastic bags, and then cooked in water at 80°C. The meat was removed when the center temperature was 70°C, cooled to room temperature and the surface water was drained. Then, the sample was weighed (W2) again. The percentage of cooking loss was calculated using the formula “(W1-W2)/W1 × 100”.

The cooked samples were cut into uniform sizes parallel to the muscle fibers and placed in a texture analyzer (TAXT2i Stable Miroc System, England) for shear force measurements, as described by Li et al. (2022a).

### Immunostaining

The breast/thigh muscles were removed separately to distinguish the muscle fiber types by immunostaining. Muscle tissue was fixed in the 4% paraformaldehyde solution. The tissues were embedded in a paraffin solution and sliced into 5 to 8 mm thick sections using a pathological microtome (RM2016, Leica, PRC). After antigen retrieval was performed, the sections were blocked by sequentially placing them in a 3% hydrogen peroxide (**H_2_O_2_**) solution and bovine serum albumin (**BSA**). Primary antibodies (heavy chain rabbit pAb for anti-fast skeletal myosin, dilution ratio of 1:5000; Service bio; heavy chain rabbit pAb for anti-slow skeletal myosin, dilution ratio of 1:500; Service bio) were added to the sections after removing the sealing solution and incubated for 12 h at 4°C. Afterward, the tissue sections were covered with HRP-labeled goat anti-rabbit IgG (dilution ratio 1:500; Service bio) or goat anti-rabbit IgG with Alexa Fluor 488 (dilution ratio 1:400; Service bio) and incubated for 50 min. Finally, the dye solution of 4′,6-diamidino-2-phenylindole (**DAPI**) was dropped into the washed slices for staining. The stained type I myofibers and type II myofibers are displayed in green and red, respectively. Statistical data related to muscle fiber types were obtained using the “ImageJ” program.

### Real-Time Quantitative PCR Detection

Total RNA was extracted according to a previously reported protocol (Cui et al., 2023). The RNA was synthesized into cDNA using an Evo M-MLV RT mix Kit without gDNA impurities for qPCR (AG, Hunan, PRC). qPCR was performed using an instrument (ABI-7500) and a SYBR Green Premix Pro Taq HS Premix (Rox Plus) (2 ×) qPCR kit (AG, Hunan, PRC). The primers listed in Table 2 were designed for RT‒qPCR by Accurate Biology. The gene expression was performed with the 2^-ΔΔCt^ method, and *β-actin was* acted as the internal reference gene.

**Table 2:** Primers used for RT‒qPCR.

Gene	Primer sequence (5′–3′)	Product size (bp)
*β-actin*	F: ATTGTCCACCGCAAATGCTTC R: AAATAAAGCCATGCCAATCTCGTC	113
*MyHC Ⅰ*	F: GCTCTCAGGGCGTAACAACT R: CAAAGTGCATGATAGCGCCC	141
*MyHC Ⅱa*	F: CAAGCATTGACCAGCTGCC R: AGATAAGGAGCAGCCTCCCC	141
*MyHC Ⅱb*	F: TCCAGTCAGCACAAGACCTTC R: GACTTTCGGAGGTAGGGAGC	131
*AMPK*	F: CTCAGAGTGCGTCGGAAGAAC R: CTGCTCCATCACCTCGTCATC	126
*SIRT1*	F: CACGCCTTGCTGTAGACTTCC R: ATGAACTTGTGGCAGAGAGATGG	148
*PGC-1α*	F: AACCAGCAGAGAACAGGAACA R: ACTCAGGTGTCAATGGAAGTGAT	126
*Nrf2*	F: TCGCAGAGCACAGATACTTCAA R: CTGGAGAAGCCTCATTGTCATCTA	109
*HO-1*	F: GTCCCGAATGAATGCCCTTGA R: ATGACCGTTCTCCTGGCTCTT	139
*CAT*	F: GGAGGTAGAACAGATGGCGTATG R: CGATGTCTATGCGTGTCAGGAT	114
*SOD*	F: CGCAGGTGCTCACTTCAATCC R: CAGTCACATTGCCGAGGTCAC	89
*GPx*	F: CAAAGTTGCGGTCAGTGGA R: AGAGTCCCAGGCCTTTACTACTTTC	136

*AMPK*: AMP-activated protein kinase; *SIRT1*: silent information regulator 1; *PGC-1α*: peroxisome proliferator-activated receptor-γ coactivator-1α; *Nrf2*: nuclear factor-like-2 factor; *HO-1*: heme oxygenase-1; *CAT*: catalase; *SOD*: superoxide dismutase; *GPx*: glutathione peroxidase.

### Western Blotting

The total protein extraction method was based on a previous method, with some modifications (Xiao et al., 2022). Briefly, the proteins (30 μg) that had been separated on SDS‒PAGE gels of the appropriate concentration were transferred to polyvinylidene difluoride membranes. The polyvinylidene difluoride membranes were then incubated with the primary antibodies as follows: AMPK (#2603, Cell Signaling Technology, 1:1000), phosphorylated-AMPK (Thr172) (p-AMPK, #2535T, Cell Signaling Technology, 1:1000), SIRT1 (#8069, Cell Signaling Technology, 1:1000), PGC-1α (#2178, Cell Signaling Technology, 1:1000), slow myosin heavy chain (MyHC, M8421, Sigma, 1:1000), fast MyHC (M4276, Sigma, 1:1000); nuclear factor-like 2 (Nrf2, 16396-1AP, Proteintech Company, Wuhan, PRC, 1:5000); catalase (CAT, 21260-1 AP, Proteintech Company, Wuhan, PRC, 1:10000); heme oxygenase 1 (HO-1, 10701-1 AP, Proteintech Company, Wuhan, PRC, 1:2000); superoxide dismutase (SOD, 24127-1 AP, Proteintech Company, Wuhan, PRC, 1:10000); glutathione peroxidase (GPx, ab125066, Abcam, Shanghai, PRC, 1:5000); and β-actin (ab6276, Abcam, Shanghai, PRC, 1:5000). Afterward, the membranes were developed with an IgG HRP-linked secondary goat anti-rabbit antibody (7074S; Cell Signaling, USA; 1:2000) or an IgG HRP-conjugated secondary goat anti-mouse antibody (GB23301; Servicebio Company, Wuhan, PRC; 1:5000). The membrane was then incubated with enhanced chemiluminescence (**ECL**) reagents and scanned using the ChemiDoc MP imaging system (Bio-Rad, Hercules, CA). The intensity of the protein bands was analyzed using Image Lab (Version 5.1, Bio-Rad).

### Enzyme Activity and Malondialdehyde Content

A 10% tissue homogenate was prepared, and the supernatant was obtained via centrifugation at 1,000 × *g* for 10 min at 4 °C to measure the antioxidant parameters. The tissue protein content, GPx, SOD, total antioxidant capacity (**T-AOC**), CAT and MDA content were assayed with reagent kits for commercial use (Nanjing Jiancheng Bioengineering Institute, Nanjing, PRC). The units of enzyme activity and T-AOC were labeled U/mg protein, while the units of the MDA content were reported as nmol/mg protein.

### Statistical Analysis

The muscle fiber composition and gene and protein expression data were analyzed using the method of comparing means (95% confidence intervals) with SPSS software (26.0, IBM-SPSS, Inc., Chicago, IL) followed by one-way analysis, and differences between treatments were detected using the least significant difference (**LSD**) post hoc test, with feed additive treatments used as dependent variables. For the other data, such as the antioxidant parameters (CAT, SOD, GPx, T-AOC, and MDA) and meat quality (pH, L*, a*, b*, drip loss, cooking loss, and shear force), 2-way ANOVA was applied with the MIXED procedure of SAS software (version 9.0). The treatment and muscle type were considered fixed factors, whereas the carcass type was a random factor. *P* < 0.05 was set up for the statistically significant difference between these data.

## RESULTS

This study was conducted to investigate the effects and underlying mechanisms of adding 2 different concentrations of BS to a broiler diet on improving muscle quality. The regulation of muscle fiber type transformation and antioxidant capacity, as well as the signaling pathways involved, were explored.

### Meat Quality

Five crucial indicators (Table 3) were analyzed to evaluate the impact of BS on the quality of meat, such as pH value, color, drip and cooking loss percentage, and shear force value.

**Table 3:** Effects of BS on the meat quality of broilers.

Items	Muscles (M)	Treatment (T)			Means	SE	*P* value		
		CON	LBS	HBS			T	M	T*M
pH_45 min_	breast	6.36	6.57	6.40	6.44	0.02	<0.01	0.79	0.06
	thigh	6.40	6.49	6.46	6.45				
	mean	6.38^b^	6.53^a^	6.43^b^		0.02			
pH_24 h_	breast	5.89	6.02	5.94	5.95^y^	0.02	<0.01	<0.01	0.98
	thigh	6.13	6.25	6.17	6.18^x^				
	mean	6.01^b^	6.13^a^	6.06^b^		0.02			
Luminance (L*)	breast	53.54^c^	51.83^d^	51.86^d^	52.41	0.32	<0.01	<0.01	0.03
	thigh	57.60^a^	54.44^c^	56.01^b^	56.01				
	mean	55.57	53.14	53.93					
Redness (a*)	breast	2.70	3.59	2.97	3.09^y^	0.10	<0.01	<0.01	0.61
	thigh	3.39	3.98	3.65	3.67^x^				
	mean	3.05^b^	3.78^a^	3.31^b^		0.12			
Yellowness (b*)	breast	10.11^b^	6.66^e^	7.08^e^	7.95	0.18	<0.01	<0.01	<0.01
	thigh	10.64^a^	8.02^d^	9.00^c^	9.22				
	mean	10.38	7.34	8.04					
Drip loss (%)	breast	2.16	1.65	1.89	1.90	0.07	<0.01	0.53	0.18
	thigh	2.32	1.80	1.74	1.95				
	mean	2.24^a^	1.72^b^	1.82^b^		0.08			
Cooking loss (%)	breast	14.79	12.15	12.44	13.13	0.39	<0.01	0.22	0.36
	thigh	13.40	12.58	11.34	12.44				
	mean	14.10^a^	12.37^b^	11.89^b^		0.48			
Shear force (N)	breast	23.44^a^	13.49^c^	18.32^b^	18.42	0.54	<0.01	<0.01	<0.01
	thigh	17.41^b^	11.29^d^	13.49^c^	14.06				
	mean	20.42	12.39	15.90					

T*M interaction indicates differences between treatments and muscles. Different letters indicate significant differences at *P* < 0.05. SE: standard error. CON: broilers fed the basal diet; BS: *Bacillus subtilis*; LBS: broilers fed 300 mg/kg BS; HBS: broilers fed 500 mg/kg BS. All data in the table represent the means ± SEs of six different animals.

Compared with the CON group, a low dosage of BS significantly increased the pH_45min_ and pH_24h_ of broiler meat (*P* < 0.05), and no change in pH was detected in the group of high-dose BS (*P* > 0.05). Significantly higher pH_45min_ and pH_24h_ values were detected after treatment with LBS than with HBS *(P* < 0.05*)*. The pH_24h_ was significant different between the muscles, and the pH_24h_ was higher in thigh muscle than in breast muscle (*P* < 0.05).

The L* and b* values was significantly downregulated by BS supplementation (*P* < 0.05), while the a* value was significantly upregulated by LBS treatment (*P* < 0.05). Compared with HBS treatment, LBS treatment significantly reduced the L* and b* values in thigh muscle (*P* < 0.05). Moreover, notable variations in meat color were observed between different parts of the body, specifically the breast and thigh muscles, with significantly higher values of L*, a*, and b* in the thigh than in the breast (*P* < 0.05).

Compared with the CON group, the addition of BS to the diet significantly reduced drip loss in broiler meat (*P* < 0.05). Nevertheless, no difference was found between the 2 BS treatments (*P* > 0.05). Differences between the thigh and the breast were also found to result in little change in drip loss (*P* > 0.05).

Dietary BS significantly reduced the degree of cooking loss (*P* < 0.05)*.* However, the degree of cooking loss was not significantly different between the BS groups (*P* > 0.05). Similarly, the differences in the cooking loss of the breast and thigh muscles were also not significant (*P* > 0.05).

Compared with HBS, LBS treatment significantly reduced the shear force of broiler meat (*P* < 0.05). Furthermore, statistical analysis revealed that the thigh muscle was more tender than the breast muscle was (*P* < 0.05). Moreover, the shear force of the thigh was lower than that of the breast, indicating potential variations in muscle fiber types (*P* < 0.05).

### Muscle Fiber Types

**Figure 1:**
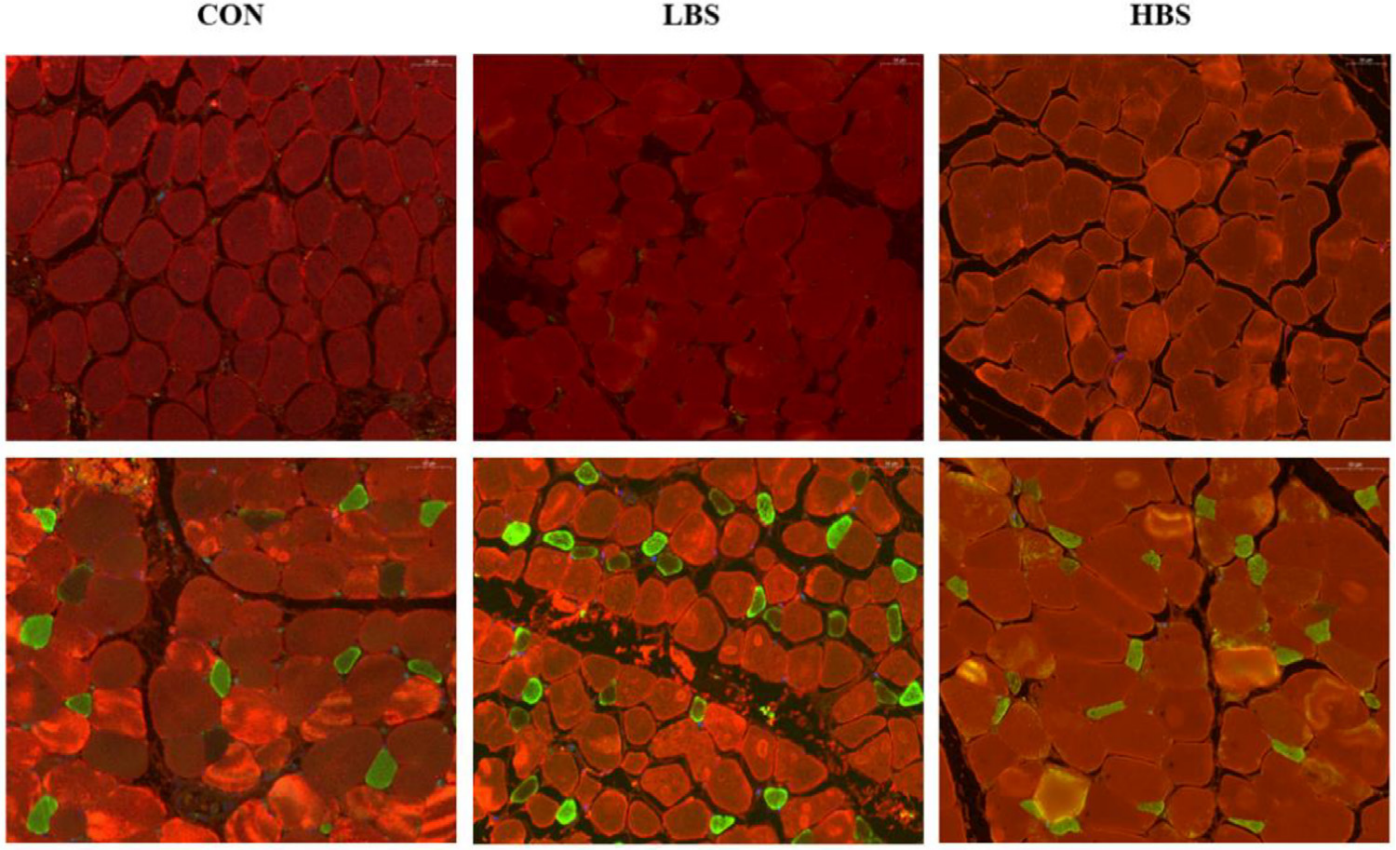
Representative cross-sections of the breast and thigh muscles of broilers. CON: broilers fed the basal diet; BS: *Bacillus subtilis*; LBS: broilers fed 300 mg/kg BS; HBS: broilers fed 500 mg/kg BS. First row of pictures: breast muscle; second row of pictures: thigh muscle. Muscle fiber types: type I, green; type II, red. Bar = 50 μm.

**Figure 2:**
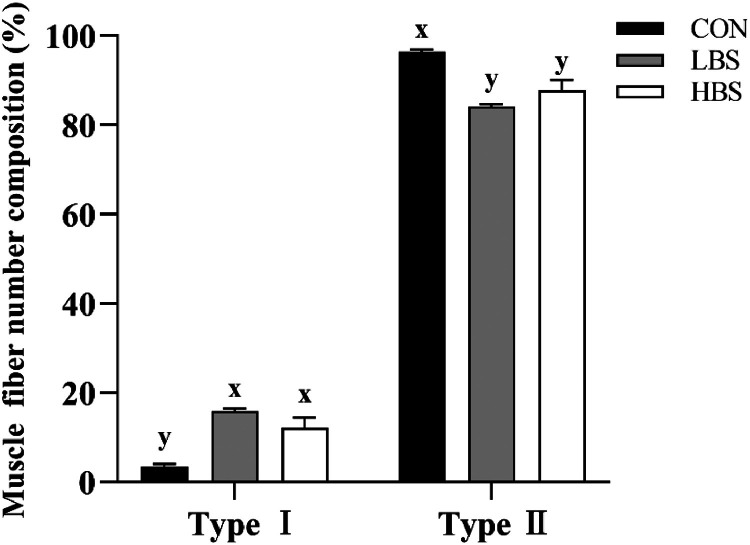
Dietary supplementation of BS affected the composition of muscle fiber types in the thigh muscle. CON: broilers fed the basal diet; BS: *Bacillus subtilis*; LBS: broilers fed 300 mg/kg BS; HBS: broilers fed 500 mg/kg BS. (x, y) indicated significant difference in muscle fiber composition among the CON group, LBS group and HBS group (*P* < 0.05).

An immunofluorescence experiment was performed to better observe the myofiber composition of the broilers. Importantly, the green staining represents type I muscle fibers, whereas the red staining represents type II muscle fibers. The findings presented in Fig. 1 illustrate the impact of BS on muscle fiber type conversion in different muscles of broilers. The experimental evidence revealed that the breast muscle of broilers exclusively comprised type II fibers and that the fiber composition was not affected by BS. In contrast, the thigh muscle of broilers contained both type I and II fibers. Notably, BS resulted in a greater proportion of type I muscle fibers. Furthermore, the results depicted in Figure 2 illustrate that the inclusion of BS in the diet influenced the distribution of type I muscle fibers in the thigh muscle, leading to a decreased proportion of type II muscle fibers (*P* < 0.05).

**Figure 3:**
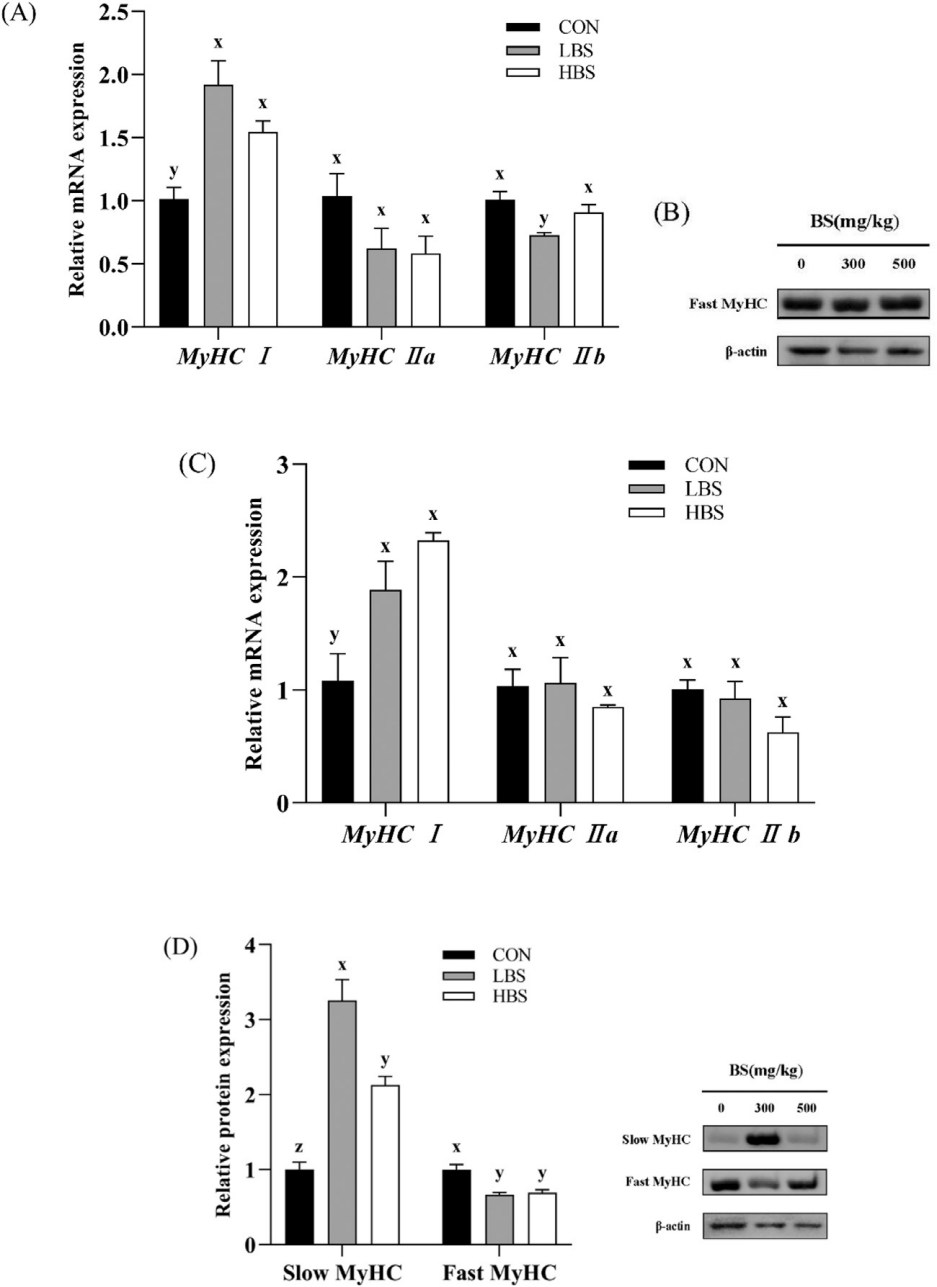
Effect of BS on muscle fiber type-related mRNA and protein expression in the breast and thigh muscles of broilers. CON: broilers fed the basal diet; BS: *Bacillus subtilis*; LBS: broilers fed 300 mg/kg BS; HBS: broilers fed 500 mg/kg BS. (A) and (B) Breast muscle, (C) and (D) thigh muscle. (x, y, z) indicate significant differences in relative mRNA or protein expression between the CON group, LBS group and HBS group (*P* < 0.05).

The relative expression levels of the MyHC I, IIa and IIb genes and proteins were determined to investigate the muscle type transformation induced by BS from a molecular point of view. Figures 3A and 3C show that both LBS and HBS treatments significantly upregulated *MyHC I* gene expression (*P* < 0.05). No significant alterations in the *MyHC Ⅱa* mRNA levels in the muscles were detected (*P* > 0.05)*.* Nevertheless, no difference of *MyHC I* mRNA levels was found between LBS and HBS groups (*P* > 0.05)*.* Additionally, the administration of a low dosage of BS led to a significant decrease in *MyHC Ⅱb* gene expression in the breast muscle compared with that in the other 2 groups (*P* < 0.05). Figure 3B shows that the expression of fast MyHC proteins was exclusively observed in the breast muscle and that the administration of BS did not significantly affect their expression (*P* > 0.05). In contrast, as shown in Figure 3D, the addition of BS to the diet significantly increased the expression of the slow MyHC protein while reducing the expression of the fast MyHC protein in the thigh (*P* < 0.05). Furthermore, significantly higher protein expression of the slow MyHC was detected in the LBS group than in the HBS group (*P* < 0.05).

**Figure 4:**
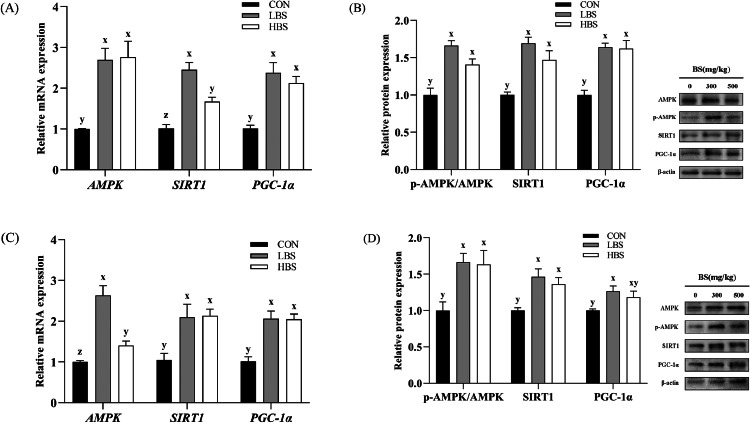
Effect of BS on the expression of AMPK/SIRT1/PGC-1α signaling pathway-related mRNAs and proteins in the breast and thigh muscles of broilers. AMPK: AMP-activated protein kinase; SIRT1: silent information regulator 1; PGC-1α: peroxisome proliferator-activated receptor-γ coactivator-1α. CON: broilers fed the basal diet; BS: *Bacillus subtilis*; LBS: broilers fed 300 mg/kg BS; HBS: broilers fed 500 mg/kg BS. (A) and (B) Breast muscle, (C) and (D) thigh muscle. (x, y, z) indicate significant differences in relative mRNA or protein expression between the CON group, LBS group and HBS group (*P* < 0.05).

The expression of the AMPK, SIRT1, and PGC-1α genes and proteins was determined to investigate the mechanisms underlying the effect of BS on the muscle fiber type transformation. Figures 4A and 4C show that BS significantly upregulated the expression of the *AMPK* gene (*P* < 0.05). The AMPK expression in thigh muscle was increased by LBS but not HBS (*P* < 0.05)*.* Similarly, BS markedly upregulated the gene expression levels of *SIRT1* and *PGC-1α* (*P* < 0.05). In the breast muscle, a higher level of the *SIRT1* mRNA was detected after treatment with LBS than after treatment with HBS (*P* < 0.05). Figures 4B and 4D shows that BS significantly upregulated the protein expression of the p-AMPK/AMPK, SIRT1, and PGC-1α (*P* < 0.05).

### Antioxidant-Related Indicators

In this study, both the gene and protein expression levels of Nrf2, HO-1, CAT, SOD, and GPx were assessed. The activities of the antioxidant enzymes CAT, SOD, and GPx, as well as the T-AOC and MDA content, were measured.

**Figure 5:**
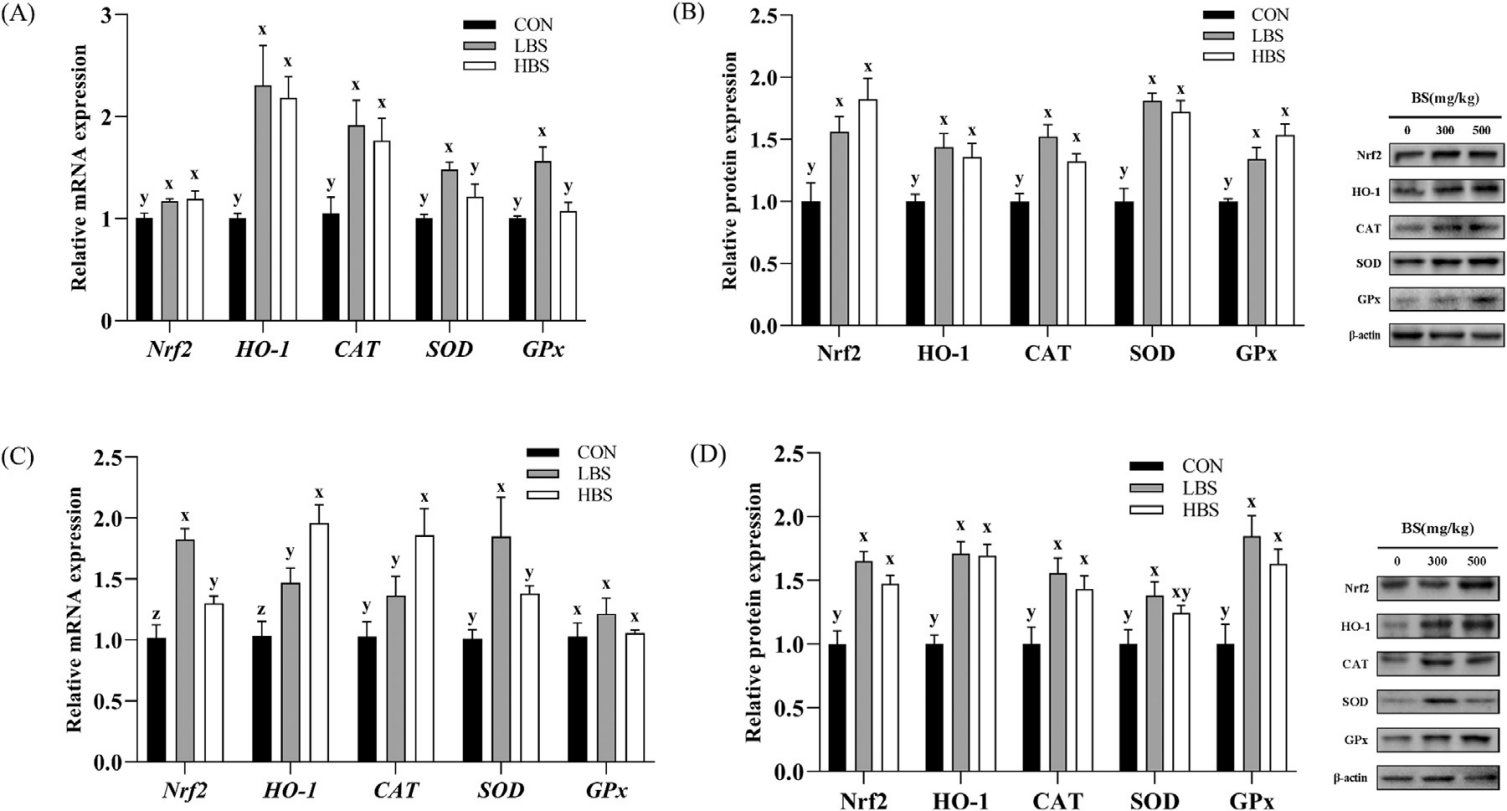
Effect of BS on antioxidant capacity-related mRNA and protein expression in the breast and thigh muscles of broilers. Nrf2: nuclear factor-like-2 factor; HO-1: heme oxygenase-1; CAT: catalase; SOD: superoxide dismutase; GPx: glutathione peroxidase. CON: broilers fed the basal diet; BS: *Bacillus subtilis*; LBS: broilers fed 300 mg/kg BS; HBS: broilers fed 500 mg/kg BS. (A) and (B) Breast muscle, (C) and (D) thigh muscle. (x, y, z) indicate significant differences in relative mRNA or protein expression between the CON group, LBS group and HBS group (*P* < 0.05).

As depicted in Figures 5A and 5C, BS significantly upregulated the expression of the *Nrf2* gene in the muscles (*P* < 0.05). The levels of the *Nrf2* mRNA were higher in thigh muscle of the LBS group than HBS group (*P* < 0.05). BS significantly increased the expression levels of the *HO-1* and *CAT* genes in the breast muscle (*P* < 0.05)*.* Compared with those in the other two groups, the *SOD* and *GPx* mRNAs levels in the LBS group was significantly upregulated (*P* < 0.05). The HBS group presented the highest expression of the *HO-1* and *CAT* genes (*P* < 0.05). The gene expression of *SOD* was significantly increased by LBS (*P* < 0.05)*.* As shown in Figures 5B and 5D, BS increased the protein expression of the muscular Nrf2, HO-1, CAT, SOD, and GPx (*P* < 0.05).

The data in Table 4 (45 min *postmortem*) indicate that BS significantly increased the GPx activity and T-AOC while reducing the MDA level in the muscles (*P* < 0.05). LBS treatment resulted in increased CAT and SOD activities than the CON group (*P* < 0.05). The GPx activity and T-AOC of thigh muscle were higher than those of breast muscle, accompanied by lower levels of MDA (*P* < 0.05). BS resulted in higher CAT activity in the thigh than in the breast (*P* < 0.05). Table 5 presents the differences in antioxidant enzyme activities in muscles at 24 h *postmortem*. The BS group presented increased CAT activities, GPx, and T-AOC, along with a decreased muscle MDA content (*P* < 0.05). GPx activity and MDA content in the thigh were significantly higher than those in the breast (*P* < 0.05). Compared with breast muscle, LBS treatment notably increased the T-AOC value of the thigh muscle (*P* < 0.05).

**Table 4:** Effects of BS on the antioxidant capacity of the breast and thigh muscles of broilers at 45 min *postmortem.*

Items (45 min)	Muscle (M)	Treatment (T)			Mean	SE	*P* value		
		CON	LBS	HBS			T	M	T*M
CAT (U/mg prot)	breast	0.94^d^	1.55^b^	0.99^d^	1.16	0.06	<0.01	0.11	<0.01
	thigh	0.66^e^	1.74^a^	1.31^c^	1.23				
	mean	0.80	1.64	1.15					
SOD (U/mg prot)	breast	6.08	7.19	7.00	6.76	0.18	0.04	0.51	0.57
	thigh	6.30	6.89	6.57	6.59				
	mean	6.19^b^	7.04^a^	6.79^ab^		0.22			
GPx (U/mg prot)	breast	75.32	101.88	83.56	86.92^y^	3.27	<0.01	0.01	0.42
	thigh	82.98	107.16	99.62	96.59^x^				
	mean	79.15^c^	104.52^a^	91.59^b^		3.69			
T-AOC (U/mg prot)	breast	0.99	1.51	1.20	1.23^y^	0.04	<0.01	0.01	0.51
	thigh	1.16	1.57	1.42	1.38^x^				
	mean	1.08^c^	1.54^a^	1.31^b^		0.05			
MDA (nmol/mg prot)	breast	0.50	0.25	0.28	0.34^x^	0.02	<0.01	<0.01	0.59
	thigh	0.38	0.17	0.16	0.23^y^				
	mean	0.44^a^	0.21^b^	0.22^b^		0.02			

CAT: catalase; SOD: superoxide dismutase; GPx: glutathione peroxidase; T-AOC: total antioxidant capacity; MDA: malondialdehyde. T*M interaction indicates differences between treatments and muscles. Different letters indicate significant differences at *P* < 0.05. SE: standard error. CON: broilers fed the basal diet; BS: *Bacillus subtilis*; LBS: broilers fed 300 mg/kg BS; HBS: broilers fed 500 mg/kg BS. All data in the table represent the means ± SEs of six different animals.

**Table 5:** Effects of BS on the antioxidant capacity of the breast and thigh muscles of broilers at 24 h *postmortem.*

Items (24 h)	Muscle (M)	Treatment (T)			Mean	SE	*P* value		
		CON	LBS	HBS			T	M	T*M
CAT (U/mg prot)	breast	0.45	0.69	0.52	0.55	0.04	<0.01	0.10	0.32
	thigh	0.49	0.72	0.73	0.65				
	mean	0.47^b^	0.70^a^	0.63^a^		0.05			
SOD (U/mg prot)	breast	6.03	6.32	6.52	6.29	0.14	0.17	0.19	0.66
	thigh	6.29	6.78	6.56	6.55				
	mean	6.16	6.55	6.54		0.17			
GPx (U/mg prot)	breast	45.45	93.73	80.29	73.16^y^	4.34	<0.01	<0.01	0.13
	thigh	83.93	100.94	98.70	94.52^x^				
	mean	64.69^b^	97.34^a^	89.49^a^		5.32			
T-AOC (U/mg prot)	breast	0.13^c^	1.03^b^	0.86^b^	0.68	0.06	<0.01	<0.01	<0.01
	thigh	0.15^c^	1.42^a^	0.93^b^	0.83				
	mean	0.14	1.23	0.90					
MDA (nmol/mg prot)	breast	0.81	0.32	0.33	0.49^y^	0.04	<0.01	0.02	0.44
	thigh	1.01	0.46	0.38	0.62^x^				
	mean	0.91^a^	0.39^b^	0.36^b^		0.04			

CAT: catalase; SOD: superoxide dismutase; GPx: glutathione peroxidase; T-AOC: total antioxidant capacity; MDA: malondialdehyde. T*M interaction indicates differences between treatments and muscles. Different letters indicate significant differences at *P* < 0.05. SE: standard error. CON: broilers fed the basal diet; BS: *Bacillus subtilis*; LBS: broilers fed 300 mg/kg BS; HBS: broilers fed 500 mg/kg BS. All data in the table represent the means ± SEs of six different animals.

## DISCUSSION

The results of the present study indicated that BS effectively regulated muscle fiber conversion from fast to slow, resulting in improved meat quality and an increased antioxidant capacity. Moreover, our findings suggest that the mechanism underlying the muscle fiber transformation mediated by BS may be associated with the AMPK/SIRT1/PGC-1α signaling pathway.

Consumers' meat purchasing decisions might be influenced by various physical properties, including color, tenderness, and water holding capacity (**WHC**). Recent studies have shown that BS, a high-quality probiotic, has a positive effect on meat quality (Dong et al., 2023a; Islam and Nishibori, 2023). The pH of meat, a critical indicator of meat quality, can affect the WHC, color, and tenderness (Suliman et al., 2023a). The incorporation of probiotics results in an increase in the pH of meat (Zheng et al., 2014). The data obtained from this study indicated noticeable differences in pH and meat color among the groups treated with different dosages of BS compared to those of the CON group. Generally, alterations in pH levels have an impact on the WHC of meat, whereby higher pH levels increase the WHC. This increase in the WHC is associated with improved tenderness and juiciness of the meat (Suliman et al., 2023b). The addition of BS to the diet in this trial resulted in a reduction in drip loss and cooking loss, ultimately increasing the WHC of broiler meat. These results are consistent with those of a previous report (Park and Kim, 2014). Shear force is an important measure of meat tenderness (Lukkananukool et al., 2023). An investigation conducted on broilers demonstrated that the dietary probiotic *Bacillus coagulans* ZJU0616 had a beneficial effect on shear force, indicating improved tenderness of the meat (Zhou et al., 2010). The present research found that BS led to a significant reduction in shear force, which led to an increase in the tenderness of the meat. In conclusion, dietary BS effectively enhanced meat quality by influencing pH levels, meat color, WHC, and shear force in the muscles. The LBS group displayed the most significant improvement in meat quality. However, supplementation with BS in the diet did not positively affect chicken meat quality in another study (Upadhaya et al., 2019). The variations in outcomes could be attributed to factors such as the amount administered, the specific bacterial strain used, the interaction with the gut microbiota, and the use of different chickens fed special diets in separate experiments.

The type of muscle fiber is closely associated with important attributes of meat quality, including pH, color, tenderness, and WHC (Lee et al., 2010; Zhang et al., 2015; Mo et al., 2023). The current data revealed significant differences in pH, meat color, and shear force values between the breast and thigh, indicating variations in chicken meat quality. These differences can be attributed to the presence of different fiber types in the respective muscles. Liu et al. (2022) reported that the administration of probiotics in feed resulted in increased expression of muscle type I fibers in the longissimus thoracis of lambs. Our study provides evidence that dietary BS can increase the expression of genes and proteins in muscle type I fibers while reducing the expression in muscle type II fibers. This enhanced the transformation of muscle fiber types, ultimately leading to improvements in meat quality.

Muscle fiber transformation is associated with various signaling pathways, among which AMPK plays a crucial role (Rockl et al., 2007). Sun et al. (2022) reported that *Bacillus natto* altered AMPK expression in obese rats. Feng et al. (2021) reported that exogenous probiotics (*Bacillus subtilis* and *Lactobacillus plantarum*) activate AMPK gene expression in perch, leading to a reduction in lipid deposition. The present data indicated that BS increased the mRNA level of AMPK and concurrently upregulated its protein expression, confirming the ability of BS to stimulate AMPK signaling. AMPK activation triggers the activation and acetylation of SIRT1, subsequently activating PGC-1α (Xu et al., 2020). Previous studies have shown that AMPK directly phosphorylates PGC-1α, a crucial factor involved in the conversion of skeletal muscle fibers from type II to type I (Lin et al., 2002; Zhang et al., 2017a; Wang et al., 2022). The present data revealed that BS increased the expression of the p-AMPK/AMPK, SIRT1, and PGC-1α proteins in muscle. This observation suggests that BS activated the AMPK/SIRT1/PGC-1α signaling pathway. In addition, type II muscle fibers transformed into type I fibers. These findings suggest a potential using of BS in facilitating the conversion of myofiber types to slow myofibers through the AMPK/SIRT1/PGC-1α signaling pathway. However, more investigations are needed to elucidate the specific mechanism of action involved.

BS has antioxidant abilities that reduce the damage caused by oxidative stress in animals (Zhang et al., 2017b; Li et al., 2022b). SOD, CAT and GPx are crucial components of the redox balance and antioxidant defense system (Yao et al., 2023). The total antioxidant capacity (T-AOC) is commonly used to evaluate antioxidant potential (Wei et al., 2019). In the present broiler experiment, BS resulted in increases in muscular CAT, SOD, and GPx activities, as well as the T-AOC, which counteracted oxidative stress and eliminated ROS produced by environmental and other stimuli. Notably, the antioxidant capacity of the thigh was generally superior to that of the breast, possibly because of the presence of type I rather than type II muscle fibers. Type I muscle fibers exhibit higher antioxidant enzyme activity than type II muscle fibers (Qaisar et al., 2016). Nrf2 is crucial for the activation of HO-1, SOD, CAT and GPx (Jin et al., 2021b; Wei et al., 2022). Activated Nrf2 stimulates the synthesis and expression of intracellular phase II metabolic enzymes and antioxidant enzymes, resulting in increased defenses against oxidative stress (Liu et al., 2014). Recent studies have provided evidence that probiotics enhance the antioxidant capacity by activating the Nrf2 signaling pathway (Elsadek et al., 2023; Lu et al., 2023). Consistent with these findings, the present study revealed alterations in the expression of the HO-1, CAT, SOD, and GPx mRNAs in response to both high and low dosages of BS, accompanied by increased protein expression. Overall, the present findings suggested that BS increased the antioxidant capacity, suppressed lipid oxidation, and improved the meat quality of broilers.

## CONCLUSIONS

In conclusion, dietary BS effectively enhanced various aspects of broiler meat quality, facilitated the conversion of myofibers to type I muscle fibers by activating the signaling pathway of AMPK/SIRT1/PGC-1α, and increased muscle antioxidant ability by activating the Nrf2 signaling pathway. Under the conditions of this experiment, the greatest improvement in broiler meat quality could be achieved with 300 mg/kg dietary BS.
